# The impact of hypophosphatemia on biochemical profile and renal outcomes in primary hyperparathyroidism: a nationwide retrospective study

**DOI:** 10.55730/1300-0144.6095

**Published:** 2025-11-04

**Authors:** Naim ATA, Bekir UÇAN, Halil DURANTAŞ, Oğulcan BOZ, Mustafa ŞAHİN, M. Mahir ÜLGÜ, Şuayip BİRİNCİ, Muhammed KIZILGÜL

**Affiliations:** 1 General Directorate of Health Information System, Turkish Ministry of Health, Ankara, Turkiye; 2 Department of Endocrinology and Metabolism, Etlik City Hospital, Ankara, Turkiye; 3 Division of Endocrinology and Metabolism, Department of Internal Medicine, Faculty of Medicine, Ankara University, Ankara, Turkiye; 4 Deputy Minister, Turkish Ministry of Health, Ankara, Turkiye; 5 Division of Diabetes, Endocrinology, and Metabolism, Department of Medicine, University of Minnesota, Minneapolis, USA

**Keywords:** Hypophosphatemia, primary hyperparathyroidism, renal stone

## Abstract

**Background/aim:**

Although low phosphorus (P) concentrations are a recognized feature of primary hyperparathyroidism (PHPT), they are not among the diagnostic or surgical criteria. The present study evaluates the association between serum phosphorus levels and clinical outcomes in PHPT patients.

**Materials and methods:**

A search of the Turkish Ministry of Health’s National Electronic Database was conducted using ICD-10 diagnostic codes and laboratory data to identify PHPT cases within the Turkish population from 2017 to 2022.

**Results:**

The records of a total of 113,330 PHPT patients (77.5% female; mean age 58.9 ± 15.6 years) were analyzed, revealing a mean serum phosphorus level of 3.24 ± 0.79 mg/dL. Patients with nephrolithiasis, vitamin D <20 μg/L, and calcium ≥11.4 mg/dL had significantly lower phosphorus levels (p<0.0001). Hypophosphatemia (HypoP) (P < 2.5 mg/dL) was present in 14.3% of patients and was associated with higher parathyroid hormone, calcium, and alkaline phosphatase levels, and lower vitamin D levels (all p < 0.0001). HypoP independently increased the risk of kidney stone formation (OR = 1.53; 95% CI 1.46–1.61).

**Conclusion:**

HypoPis associated with more severe biochemical abnormalities and a greater prevalence of nephrolithiasis in PHPT. In regions where vitamin D deficiency is common, low phosphorus levels may indicate more severe diseases, and so routine phosphorus monitoring should be considered as part of PHPT management.

## 1. Introduction

Primary hyperparathyroidism (PHPT) is a common endocrinological disorder characterized by elevated serum calcium concentrations alongside increased or aberrantly normal levels of parathyroid hormone (PTH), attributable to hyperactivity of the parathyroid glands. In PHPT cases, serum phosphorus (P) levels are typically found to be low or within the lower range of normal. A significant proportion of patients diagnosed with PHPT exhibit mild hypophosphatemia (HypoP) [1]. Parathyroid hormone (PTH) enhances the excretion of phosphate by suppressing the functioning of sodium-phosphate cotransporters [2]. The primary mechanism responsible for HypoP in PHPT is increased urinary excretion of phosphate [3]. In the diagnosis and management of PHPT, calcium and PTH levels are given priority over phosphorus levels. It should be noted that hypoP is often overlooked during the diagnostic process for PHPT, and there is a lack of relevant data in the existing literature.

The main complications associated with PHPT affect the kidneys and bones, although kidney stones are reported in less than 20% of PHPT patients, and bone disease is reported in even fewer cases [4]. The likelihood of progression to bone or kidney disease appears to be unaffected by hypercalcemia severity [5].

A relationship has been identified between serum P levels and PTH production, with a possible indirect effect on calcium levels [6]. HypoP was detected in approximately 10%–20% of the PHPT cases investigated in a large series [7]. There have been two studies to date reporting significantly lower serum P levels in PHPT patients with kidney stones [8,9]. In a study of 472 patients with PHPT, low phosphorus levels in asymptomatic patients with PHPT were identified as a possible indicator for surgery, regardless of age and hypercalcemia degree [10]. In a more recent study, the risk of osteoporosis and nephrolithiasis was stated to be higher in PHPT patients with low P levels [11].

In the present study we investigate the influence of phosphorus levels on clinical outcomes in cases with PHPT through an evaluation of a large population database.

## 2. Materials and methods

### 2.1. Study population and design

All procedures conducted in the study were carried out in accordance with the principles set out in the Declaration of Helsinki, and the study protocol was approved by the Investigation Review Board under the Bioethics Committee of the General Directorate of Health Services of the Turkish Ministry of Health (IRB number: 95741342-020).

### 2.2. Data collection

The Turkish Ministry of Health National Electronic Database was accessed for the collection of study data. The Turkish Ministry of Health has hosted a national electronic database since 2016, combining all the existing alternative databases and their records of procedures, laboratory findings, drugs, and mortality data. For the present study, the records of more than 85 million members of the public who applied to the hospital between January 1, 2017, and December 31, 2022 were evaluated. In this nationwide, registry-based retrospective observational study, patients with primary hyperparathyroidism (E21) and renal stones (N20) were identified based on the International Statistical Classification of Diseases and Related Health Problems (ICD-10) codes. Laboratory variables including PTH, calcium, P, creatinine, ALP, 25OHD, and albumin were accessed from the National Electronic Database. All laboratory values measured when E21 was first diagnosed between 2017 and 2022 were recorded. Since the acquired data is from the time of initial diagnosis, it can be assumed to be the situation before medical or surgical treatment. Parameters including birth date and sex were also recorded.

High calcium levels in the presence of improperly normal or increased PTH levels were used for the diagnosis of PHPT [12]. PTH, calcium, P and creatinine level outliers were excluded. As pathology reports that could confirm the diagnosis of the patients were not available, patients with PTH levels <65 pg/mL and calcium levels of 10.4 mg/dL were excluded from the study to increase the diagnostic certainty. Colorimetric methods were used for the measurement of the serum albumin, calcium, P, and creatinine levels. Intact PTH assay (chemiluminescent immunoassay with an Immulite 2000 device; normal range 12–65 ng/L) was used for the measurement of PTH, while a radioimmune assay was used for the measurement of 25OHD levels. HypoP, described as a P level of less than 2.5 mg/dL, is categorized into three groups as mild, moderate, and severe (2–2.5 mg/dL, 1–1.99 mg/dL and <1 mg/dL, respectively) [11]. The glomerular filtration rate (GFR) was calculated using the CKD-EPI creatinine equation:


141×min(SCr/κ,1)α×max(Scr/κ,1)×1.209×0.993Age×1.018 [if female]×1.159 [if black]

where SCr is serum creatinine (in mg/dL), k is 0.7 for women and 0.9 for men, α is −0.329 for women and −0.411 for men, min is the minimum of SCr/k or 1, and max is the maximum of SCr/k or 1 [13]. Patients with a GFR below 45 were excluded from the study to exclude secondary hyperparathyroidism and phosphorus retention associated with chronic kidney disease [14].

### 2.3. Statistical analysis

JMP 16.0.1 software (SAS Institute, Cary, NC, USA) was used for the statistical analysis of the data. Continuous data were presented as mean ± standard deviation (SD) values and categorical data as numbers (n) and percentages (%).Kolmogorov–Smirnov or Shapiro–Wilk W tests were used for the evaluation of the normality of distribution. Categorical variables were analyzed using Chi-square or Fisher’s exact tests. The student’s t-test was used for the analysis of normally distributed continuous variables, while the Mann–Whitney U Test was used for nonnormally distributed variables. A Pearson correlation analysis was conducted to identify correlations between variables. A value of p < 0.05 was accepted as statistically significant. Logistic regression was performed to evaluate whether predictive variables, such as specific laboratory findings, were able to significantly predicted the development of renal stone.

## 3. Results

A retrospective analysis of a total of 113,330 patients with PHPT revealed 87,759 (77.5%) to be female and 25,571 (22.5%) to be male, with a mean age of 58.9 ± 15.6 years. Kidney stones were identified in 12,733 (11.2%) patients. The mean P level of the entire study population was 3.24 ± 0.79 mg/dL (Table 1).

Comparisons of P levels based on demographic and clinical features are presented in Table 2. Female patients had higher P levels than their male counterparts (3.31 ± 0.8 vs. 3.24 ± 0.93, p < 0.0001). Patients with kidney stones, 25OHD levels < 20 μg/L, calcium levels ≥ 11.4 mg/dL and those younger than 50 years were found to have decreased phosphorus levels (p < 0.0001). Calcium/phosphorus ratios in excess of 3.5 were observed in 43% of the patients, and a PFindex (Ca*PTH/P) of > 34 was observed in 33% of the patients.

HypoP was determined in 16,316 of the total 113,330 patients (14.3%), with a mean serum P level of 2.12 ± 0.29 mg/dL. HypoP patients had increased PTH, calcium and ALP levels and lower creatinine and 25OHD levels (p < 0.0001). HypoP was more common in male patients (29.8% vs. 21.2%, p < 0.0001), and HypoP patients were at greater risk of developing kidney stones (16.3% vs.10.4%, p < 0.0001) (Table 3).

Mild hypoP was identified in 12,180 (75%) patients, and moderate hypoP in a further 4018 (25%). Severe hypoP was observed in 118 patients; however, due to the small sample size, this group was not included in the statistical analysis. Patients with moderate hypoP were noted to be younger and to have increased PTH, calcium, creatinine, and ALP levels than those with mild hypoP. The moderate group had lower levels of 25OHD and included more male patients. The prevalence of kidney stone was greater in the moderate hypoP group than in the mild hypoP group (17.9% vs. 15.8%, p = 0.002) (Table 3).

The correlation analyses revealed P levels to be negatively correlated with PTH, alkaline phosphatase, and serum total Ca level, and positively with creatinine and 25OHD levels (p < 0.0001) (Table 4). A multivariate logistic regression analysis performed with the presence of renal stones as the dependent variable, and sex, age, serum calcium, serum phosphorus, 25-hydroxyvitamin D (25OHD), and glomerular filtration rate (GFR) as independent variables. The analysis revealed that sex (ref: male; OR: 1.47, 95% CI: 1.41–1.53, p < 0.0001), age (ref: < 50 years; OR: 1.14, 95% CI: 1.09–1.19, p < 0.0001), hypercalcemia (ref: calcium < 11.4 mg/dL; OR: 1.38, 95% CI: 1.31–1.45, p < 0.0001), and HypoP (ref: phosphorus ≥ 2.5 mg/dL; OR: 1.53, 95% CI: 1.46–1.61, p < 0.0001) were significantly associated with an increased likelihood of renal stone formation. In contrast, 25OHD (ref: < 20 ng/mL) and GFR (ref: < 60 mL/min/1.73 m^2^) were not found to be significant predictors in the multivariate model (Table 5). The predictive value of phosphate levels for the presence of renal stones was evaluated through a Receiver Operating Characteristic (ROC) analysis, and was found to be statistically significant (AUC = 0.569 95%CI: 0.467–0.750 p < 0.001) (Figure). A phosphate cut-off value of 3.07 predicted the presence of renal stones with 51% sensitivity and 60% specificity, corresponding to a Youden index (J) of 0.11, compared to 24% sensitivity and 84% specificity when a cut-off value of 2.5 was applied, yielding a Youden index (J) of 0.08. It was thus concluded that the overall diagnostic performance was relatively low for both thresholds, and that phosphate levels alone have minimal clinical utility as a predictive marker for renal stone formation.

## 4. Discussion

In this study examining the association between serum phosphorus (P) levels, laboratory parameters, and renal outcomes in patients with PHPT, hypoP was found to be associated with a higher prevalence of kidney stones in PHPT patients. Furthermore, the patients with hypoP were found to have increased serum PTH, calcium, and ALP levels and decreased 25OHD levels.

The low phosphorus levels seen in PHPT are generally moderate in severity due to decreased renal phosphorus absorption, and are balanced by the mobilization of P from bone and enhanced intestinal retention. As a result, P levels rarely fall below 2.0 mg/dL [15]. Low serum phosphorus in a patient with hypercalcemia is an important indicator of primary hyperparathyroidism, while moderately normal or low serum phosphorus levels suggest PHPT [16]. Moreover, the presence of hypoP alone in the absence of increased calcium levels may be an early sign of PHPT [17]. PHPT assessments are characteristically based on serum calcium and PTH measurements, while P levels are often ignored [18]. Although P levels do not play an important role in the diagnosis and management of PHPT, the disorder itself, which affects bone and kidney, may influence serum phosphorus concentrations. Given the prevalence of the condition and the common lack of noticeable symptoms, the existence of markers believed to correlate with disease severity is significant.

Previous animal studies have suggested that parathyroid hormone (PTH) may stimulate FGF-23 production. In PHPT, the increase in serum FGF-23 levels is thought to originate from bone. The simultaneous elevation of FGF-23 and PTH may decrease serum phosphorus levels, thereby protecting against ectopic calcification and tissue damage by reducing the calcium-phosphate product in the presence of hypercalcemia [19].

In a study of 472 patients with PHPT examining the association between serum phosphorus (P) levels and clinical outcomes, 199 (41.9%) had HypoP (<2.5 mg/dL), including 168 (84.9%) with mild and 30 (15.1%) with moderate forms. It was found that patients with hypercalcemia and elevated PTH levels were at greater risk of Hypop and kidney stones, but not osteoporosis. The authors suggested that moderate Hypop may serve as a simple, inexpensive, and accessible indicator of asymptomatic PHPT in patients, and as a potential marker for surgical intervention. Another study with a similar design reported Hypop to be associated with a higher likelihood of both osteoporosis and nephrolithiasis in PHPT [11]. The study reported a link between moderate Hypop and nephrolithiasis and osteoporosis, even in asymptomatic patients, and identified it as a potential new surgical indication criterion.

In our nationwide study of 126,454 PHPT patients, we also evaluated the relationship between serum phosphorus levels and clinical outcomes, revealing Hypop to be associated with impaired renal function and a higher prevalence of kidney stones. Collectively, these results underline the potential clinical relevance of serum phosphorus levels in the management of PHPT.

More severe Hypop and higher calcium and PTH levels are expected in PHPT patients with kidney stones [20]. Both Castellano et al. and Duger et al. report increased levels of calcium and PTH and decreased P levels in patients with nephrolithiasis [11]. These findings concur with those of the present study, in which patients with nephrolithiasis were identified with increased PTH and calcium levels, and decreased P levels.

Serum P is generally in the low–normal range in PHPT, and roughly a quarter of patients have below-normal values. Phosphorus levels above 3.5 mg/dL are not a highly anticipated finding unless kidney failure is present [1]. Evaluating P levels together with calcium values may be useful in terms of supporting diagnosis. The calcium/phosphorus ratio can be proposed as a new diagnostic index for the identification of patients with suspected PHPT. Values above 3.5 are reported to be 89% sensitive and 91% specific in the diagnosis of both classical and normocalcemic PHPT [21,22]. In the PFindex developed by Guo et al. (calculated as serum calcium x PTH/serum phosphorus), a value of > 34 was stated to discriminate between PHPT and secondary hyperparathyroidism associated with 25OHD deficiency with a sensitivity of 96.9% and specificity of 97.6% [23]. In the present study, a calcium/phosphorus ratio of >3.5 was noted in 42% of patients, and a PFindex of >34 in 34% of patients. Based on these results, the stated rates may not be as sensitive as previously thought in the diagnosis of PHPT.

Hypophosphatemia was a common finding in patients with PHPT in the studies of both Castellano et al. and Düger et al., and was associated with higher PTH and calcium levels, as well as a higher prevalence of renal stones [10,11]. Similarly, in the present study, patients with Hypop had higher PTH, calcium, creatinine, and ALP levels, and an increased rate of renal stones than those with normal phosphate levels. PHPT patients with low 25OHD levels are more likely to have hypoP [24]. Concurring with both studies, lower 25OHD levels were associated with Hypop also in the present study. Düger et al. reported further that patients with 25OHD levels below 20 μg/L were at greater risk of Hypop, which aligns with the lower 25OHD concentrations in patients with Hypop identified in the present study, particularly in those with moderate forms.

As reported by Castellano et al. and Düger et al., male patients exhibited lower serum phosphate levels, which may partly be explained by the influence of sex hormones on the biochemical manifestations of PHPT [25]. Similarly, phosphate levels were lower in the male patients than in the female patients in the present study, consistent with the findings of the earlier reports.

In the present study, the mean 25OHD value was 17.6 μg/L, and > 50% of patients had 25OHD deficiency; in contrast to PHPT patients in Western nations and the United States, where 25OHD levels are much higher and most patients are asymptomatic [26,27]. A metanalysis of 40 studies involving a total of 111,582 patients reported a high rate of 25OHD deficiency in Türkiye, which varies from 58.9% to 66.6% with a 95% CI [28]. An earlier study conducted in Türkiye reported 25OHD deficiency in 63.8% of the cases and a median 25OHD level of 16.6 μg/L in patients with PHPT, which are comparable to the values reported in our study [29].

This study had some limitations that should be considered, primarily the lack of surgical (parathyroidectomy) and pathology data confirming the PHPT diagnoses. For this reason, only patients with high PTH and calcium levels were included in the study. Secondly, since the Ministry of Health only started compiling data in 2016, data from the period before then could not be acquired as the whole country was not represented. Thirdly, we had no access to the osteoporosis and fracture histories of the patients.

A further limitation was the lack of information of the phosphorus intake of the patients, any phosphate-binding antacids, thiazide diuretics, or vitamin D supplements they were taking, or secondary causes of hyperparathyroidism. An additional limitation of our study relates to the possible coding or measurement errors that are inherent in electronic health records, as well as the single measurement of GFR without longitudinal follow-up. Finally, no bone densitometry or 24-h urine calcium records were available, which are important criteria for surgical indication in PHPT. The main strength of the present study is its large sample size, which is considerably higher than in previous reports. It is well recognized that studies based on electronic health records face certain challenges, as such databases are designed primarily for administrative and reimbursement purposes, and this is a limitation that applies to most similar studies in the literature. In Türkiye, however, the effects of this issue are relatively low, as all members of the public are covered by a unified national health insurance system under the Social Security Administration. Furthermore, as the Ministry of Health integrates ICD-10 codes with comprehensive datasets – including medication use, hospital admissions, and payment records –any inconsistencies that arise can be verified when necessary.

In conclusion, the evaluation of calcium and phosphorus levels together is essential for the accurate diagnosis and management of PHPT, as both are regulated by PTH. In countries such as Türkiye, where 25-hydroxyvitamin D deficiency is common, lower serum phosphorus levels are associated with more pronounced biochemical abnormalities and renal complications in PHPT. Patients with Hypop (HypoP) tend to exhibit higher PTH, calcium, and alkaline phosphatase (ALP) levels, and lower 25-hydroxyvitamin D concentrations. These findings suggest that serum phosphorus levels may serve as an additional biochemical parameter for risk stratification and surgical consideration in patients with primary hyperparathyroidism.

## Figures and Tables

**Figure:**
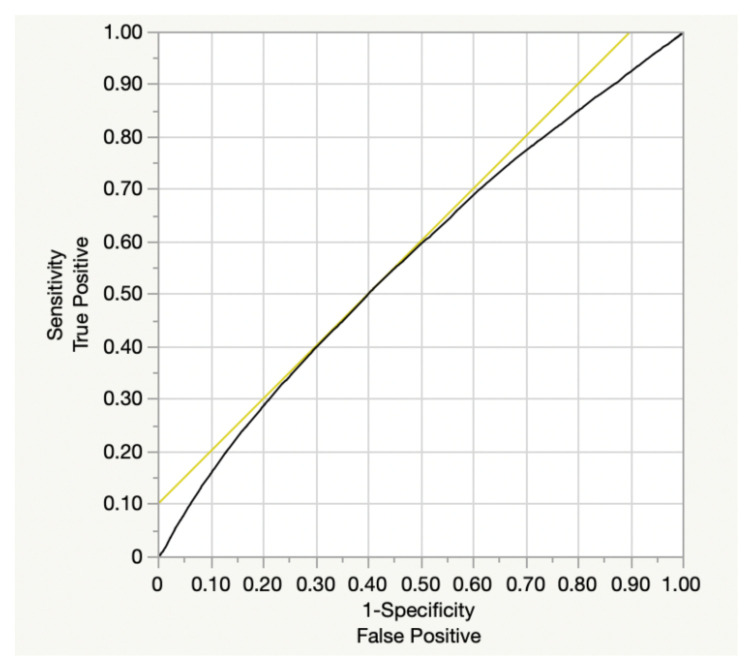
ROC curve of serum phosphorus for predicting nephrolithiasis. **Abbreviations:** ROC, receiver operating characteristic.

**Table 1:** Baseline characteristics of patients with PHPT (n:113,330).

	Mean or n	SD or (%)
**Sex (Female)**	87,759	77.4
**Age (years)**	57.51	15.36
**Calcium (mg/dL)**	10.93	0.78
**Corrected calcium (mg/dL)**	10.63	0.9
**Phosphorous (mg/dL)**	3.24	0.79
**Parathyroid hormone (pg/mL)**	125.49	92.65
**Creatinine (mg/dL)** *	0.82	0.22
**Albumin (mg/dL)** **	4.36	0.48
**Alkaline phosphatase (U/L)** ***	93.84	47.14
**25OHD (ng/mL)** ****	17.61	13.57
**Presence of renal stone**	12733	11.2

* Data available for 112.958 patients

** Data available for 106.531 patients

*** Data available for 107.702 patients

**** Data available for 96.992 patients

**Abbreviations:** PHPT, primary hyperparathyroidism; PTH, parathyroid hormone; ALP, alkaline phosphatase; GFR, glomerular filtration rate; 25OHD, 25-hydroxyvitamin D.

**Table 2:** Serum phosphorus levels by demographic and clinical characteristics.

		Mean ± SD	p
**Sex**	Female (n:87759)	3.27 ± 0.77	**<.0001**
	Male (n:25571)	3.16 ± 0.85	
**Renal stones**	Yes (n:12733)	3.09 ± 0.79	**<.0001**
	No (n:100597)	3.26 ± 0.79	
**25OHD deficiency (<20 μg/L)** *	Yes (n:67270)	3.21 ± 0.79	**<.0001**
	No (n:30098)	3.29 ± 0.75	
**Calcium ** **^3^** ** 11.4mg/dL**	Yes (n: 13250)	2.95 ± 0.99	**<.0001**
	No (n:100080)	3.28 ± 0.76	
**GFR < 60 mL/min/1.73 m** **^2^** **	Yes (n:15931)	3.36 ± 0.82	**<.0001**
	No (n:96166)	3.23 ± 0.78	
**Age younger than 50 years**	Yes (n:32953)	3.24 ± 0.85	**0.035**
	No (n:80377)	3.25 ± 0.77	

* Data available for 97,368 patients

** Data available for 112,097 patients

**Abbreviations:** PHPT, primary hyperparathyroidism; PTH, parathyroid hormone; ALP, alkaline phosphatase; GFR, glomerular filtration rate; 25OHD, 25-hydroxyvitamin D.

**Table 3:** Comparison of PHPT patients with and without hypophosphatemia, and by severity.

	P > 2.5 mg/dL (n = 97014)		P ≤ 2.5 mg/dL (n = 16316)		p
	Mean ± SD		Mean ± SD		
**Age (years)**	57.48	15.36	57.68	15.34	**0.002**
**PTH (ng/L)**	114.45	74.48	192.04	147.44	**<.0001**
**Calcium (mg/dL)**	10.89	0.76	11.20	0.86	**<.0001**
**Corrected calcium (mg/dL)**	10.58	0.87	10.98	0.96	**<.0001**
**Creatinine (mg/dL)**	0.82	0.22	0.81	0.23	**<.0001**
**25OHD (μg/L)**	17.94	13.77	15.65	12.17	**<.0001**
**ALP**	91.41	44.25	106.17	58.09	**<.0001**
**Female Gender, n (%)**	76,295(78.8)		11464 (70.2)		**<.0001**
**Renal stones, n (%)**	10,069 (10.4)		2664 (16.3)		**<.0001**
	**Mild HypoP (n =12,180)**		**Moderate HypoP (n = 4018)**		**p**
	**Mean**	**SD**	**Mean**	**SD**	
**Age (years)**	58.08	15.11	56.66	15.87	**<.0001**
**PTH (ng/L)**	177.56	129.83	237.76	184.93	**<.0001**
**Calcium (mg/dL)**	11.14	0.78	11.20	1.02	**<.0001**
**Corrected calcium (mg/dL)**	10.9	0.88	11.19	1.11	**<.0001**
**Creatinine (mg/dL)**	0.80	0.23	0.80	0.24	**0.012**
**25OHD (μg/L)**	15.77	11.93	15.24	12.83	**0.035**
**ALP**	103.71	53.88	118.27	72.14	**<.0001**
**Female Gender, n (%)**	8705 (71.5)		2683 (66.8)		**<.0001**
**Renal stones, n (%)**	1929 (15.8)		722 (17.9)		**0.002**

**Abbreviations:** PHPT, primary hyperparathyroidism; HypoP, hypophosphatemia; PTH, parathyroid hormone; ALP, alkaline phosphatase; GFR, glomerular filtration rate; 25OHD, 25-hydroxyvitamin D.

**Table 4:** Correlations between serum phosphorus and biochemical parameters.

	r	p
**Parathyroid hormone (pg/mL)**	−0.2515	<.0001
**Calcium (mg/dL)**	−0.0879	<.0001
**Corrected Calcium (mg/dL)**	−0.096	<.0001
**Creatinine (mg/dL)**	0.0572	<.0001
**25OHD (ng/mL)**	0.0614	<.0001
**Alkaline phosphatase (U/L)**	−0.0890	<.0001

**Abbreviations:** PHPT, primary hyperparathyroidism; PTH, parathyroid hormone; ALP, alkaline phosphatase; GFR, glomerular filtration rate; 25OHD, 25-hydroxyvitamin D.

**Table 5:** Results of multivariate and univariate logistic regression analyses.

	Multivariate		Univariate	
Variables	OR (95% CI)	p	OR (95% CI)	p
**Gender**, *ref: Male*	1.47 (1.41–1.53)	<.0001		
**Age**, *ref:<50*	1.14 (1.09–1.19)	<.0001		
**Hypercalcemia** *ref; calcium <11.4 mg/dL*	1.38 (1.31–1.45)	<.0001		
**Hypophosphatemia**, *ref: >2.5 mg/dL*	1.53 (1.46–1.61)	<.0001	1.68 (1.61–1.76)	<.0001

**Abbreviations:** PHPT, primary hyperparathyroidism; HypoP, hypophosphatemia; PTH, parathyroid hormone; ALP, alkaline phosphatase; GFR, glomerular filtration rate; 25OHD, 25-hydroxyvitamin D; OR, odds ratio; CI, confidence interval; ref, reference category.
